# Profiling of B-Cell Factors and Their Decoy Receptors in Rheumatoid Arthritis: Association With Clinical Features and Treatment Outcomes

**DOI:** 10.3389/fimmu.2018.02351

**Published:** 2018-10-11

**Authors:** Javier Rodríguez-Carrio, Mercedes Alperi-López, Patricia López, Francisco J. Ballina-García, Ana Suárez

**Affiliations:** ^1^Area of Immunology, Department of Functional Biology, Faculty of Medicine, University of Oviedo, Oviedo, Spain; ^2^Instituto de Investigación Sanitaria del Principado de Asturias, Oviedo, Spain; ^3^Department of Rheumatology, Hospital Universitario Central de Asturias, Oviedo, Spain

**Keywords:** BLyS, APRIL, B-cell factors, arthritis, personalized medicine

## Abstract

**Introduction:** B-cell activation is pivotal in rheumatoid arthritis (RA) pathogenesis and represents a relevant therapeutic target. The main aim of this study was to characterize the profiles of B-cell factors and their decoy receptors in RA and evaluate their clinical relevance.

**Methods:** sBLyS, sAPRIL, sBCMA, sTACI, sBLyS-R, and several cytokines' serum levels were measured by immunoassays in 104 RA patients and 33 healthy controls (HC). An additional group of 42 systemic lupus erythematosus (SLE) patients were enrolled as disease controls. Whole blood IFI44, IFI44L, IFI6, and MX1 gene expression was measured and averaged into an IFN-score. BLyS membrane expression (mBLyS) was assessed on blood cell subsets by flow cytometry.

**Results:** increased sAPRIL and sBCMA levels were found in RA, whereas BLyS was elevated in very early RA (VERA). No differences were observed for sTACI and sBLyS-R. An increased sBLyS/sBLyS-R ratio was associated with poor clinical outcome at 6 and 12 months in VERA, whereas a positive association with disease activity was observed in established disease. Increased mBLyS expression was found on monocytes, mDCs, neutrophils and B-cells in RA, to a similar extent that in SLE patients. Cluster analysis identified a specific B-cell factors profile overrepresented in RA and associated with autoantibodies, elevated proinflammatory cytokines (IFNα, MIP1α, TNFα, IL-37, and GM-CSF) and increased type-I IFN signature. Increasing sBCMA and sBLyS serum levels upon treatment and mBLyS expression at baseline on monocytes and mDCs, but not B-cells, were associated with poor clinical outcome upon TNFα-blockade.

**Conclusions:** profound and complex alterations of soluble and membrane-bound B-cell factors are observed in RA associated with clinical outcomes, thus supporting its applicability to guide patient stratification along disease course.

## Introduction

As main orchestrators of the humoral immune response, the role of B-cells in rheumatoid arthritis (RA) has been largely investigated. Although classically considered as antibody-producing cells, the B-cell compartment is now recognized to be composed by different cell subsets at distinct differentiation stages (1). Of note, B-cell depletion therapy revealed a differential effect on distinct subsets and autoantibody levels, thus supporting that the role for B-cells goes beyond autoantibody production to include antigen presentation, T-cell activation and cytokine production (2, 3). Therefore, the control of B-cell activation and function is of pivotal relevance in RA pathogenesis.

B-cell activation is regulated by several members of the Tumor Necrosis Factor (TNF) superfamily. This family includes two closely related stimulating factors, BLyS (B Lymphocyte Stimulating factor) and APRIL (A Proliferation-Inducing Ligand), both central players of B-cell development and homeostasis (4). Altered serum levels of these cytokines have been found in immune-mediated rheumatic conditions (5). These B-cell activating factors develop their cellular responses via three different receptors: BCMA (B-cell Maturation Antigen), TACI (Transmembrane Activator and CALM Interactor) or BLyS-R (also known as BAFF receptor or BR3) (6). Recent studies have found that these receptors are present in both membrane-bound and soluble isoforms, as decoy receptors. However, their role in pathogenesis is far from being clear. Moreover, these receptors are able to bind the different B-cell factors with different affinities (6–8), hence delineating a complex network of regulatory mechanisms, with potential different cytokine-receptor axes involved, underlying B-cell activation. More importantly, different cell populations other than B-cells have been reported to produce BLyS (9, 10). All these lines of evidence support an intriguing biological complexity of the B-cell factors network. Additionally, recent studies by our group (11, 12) and others (13, 14) point toward a connection between BLyS and type I IFN in systemic conditions. However, although type I IFNs have been associated with clinical outcomes in RA (15), including ACPA levels, whether a link between type I IFN and B-cell factors exists in RA remains unknown.

B-cells, and B-cell activation, are attractive disease targets and several strategies and specific drugs have been tested in RA. Unfortunately, some of these drugs have shown a moderate clinical efficacy in clinical trials [reviewed in (16)]. Such a complex regulation within the B-cell compartment, with synergistic and redundant effects, could be a limitation for the clinical efficacy of these therapies. However, a knowledge gap exists regarding the broad picture of the B network depicted by B-cell factors and their receptors in RA. Therefore, a better understanding of the latter is crucial to ensure clinical efficacy and the identification of biomarkers to predict clinical response.

We hypothesize that different patterns among B-cell factors and their decoy receptors may be found in RA in association with clinical features. The main aim of the present study is to comprehensively analyse the network of B-cell factors in RA, including their soluble and membrane-bound forms as well as their decoy receptors, with a special focus on its clinical relevance as potential biomarkers.

## Materials and methods

### Ethics statement

The study protocol was approved by the Institutional Review Board (Comité de Ética de Investigación Clínica del Principado de Asturias, ref. PI16/00113) in compliance with the Declaration of Helsinki. All study subjects gave written informed consent.

### Patients and controls

A total of 104 patients were recruited from the Department of Rheumatology at Hospital Universitario Central de Asturias (Spain). A complete clinical examination, including Disease Activity Score 28-joints (DAS28) and HAQ calculations, was performed on all patients during their clinical appointments. Patients fulfilling the 2010 ACR/EULAR classification criteria for rheumatoid arthritis with a disease duration longer than 6 months since diagnosis were classified as established RA (*n* = 85). Patients recruited at disease diagnosis (i.e., the same day that the definite diagnosis is set) were classified as very early rheumatoid arthritis (VERA) patients (*n* = 19). VERA patients were not previously exposed to any treatment and all had a duration of symptoms below 18 weeks (mean ± SD: 12.1 ± 5.0 weeks). These patients were prospectively followed-up for 1 year, and clinical outcomes were registered at 6 (T6) and 12 (T12) months. Clinical management was performed according to EULAR recommendations (17) and clinical response was evaluated by EULAR criteria (18). Patients exhibiting a good response were considered as responders, whereas those with moderate or no response were classified as non-responders. Treatment adherence was monitored by our internal procedures in our in-day hospital (bDMARDs) and rheumatology department (csDMARDs). An additional group of 13 biological-naïve RA patients (12 women, age, median(range): 43(30–65), DAS28 5.08(1.93), 38.5% RF+, 46.1% ACPA+), candidates for TNFα-blockade was prospectively followed-up for 3 months. A blood sample was obtained before and 3-months after initiation of TNFα-blockade. Sample size calculation for this group was performed to allow the identification of a difference of 1.2 points in the DAS28 score, with an a = 0.05 and a power of 0.80 in a paired design.

A group of 33 gender- and age-matched healthy volunteers was simultaneously recruited as healthy controls (HC) from the same population. An additional group of 42 systemic lupus erythematosus patients was recruited as disease controls (Supplementary Table 1). SLE diagnosis was defined according to the ACR revised criteria for SLE classification (19). A blood sample was collected from all individuals by venipuncture. Fresh blood was processed immediately for flow cytometry experiments and RNA stabilization, and serum samples were frozen at−80°C until cytokine measurements.

### Cytokine quantification

Serum levels of circulating cytokines were analyzed by commercial immunoassays following manufacturer instructions as follows: sBLyS [Human BAFF instant ELISA, eBioscience, detection limit (d.l.) = 0.13 ng/ml], sBCMA (TNFRSF17 ELISA kit, Raybiotech, d.l. = 0.025 ng/ml), sTACI (Human TACI ELISA kit, Raybiotech, d.l. = 0.32 pg/ml), sAPRIL (Human APRIL Platinum ELISA, eBioscience, d.l. = 0.19 ng/ml), sBLyS-R (Human TNFRSF13C, abcam, d.l. = 0.78 pg/ml), IL-10 (Human IL-10 ELISA high sentivity, eBioscience, d.l. = 0.19 pg/ml), IL-37 (Human IL-37/IL1F7 ELISA, BosterBio, d.l. = 4.8 pg/ml), TNFα (Human TNFα Mini EDK, Peprotech, d.l. = 3.9 pg/ml) and IFNg (IFNg OptEIA kit, BD Bioscience, d.l. = 0.58 pg/ml). Levels of IFNα, MIP1α, IL-8 and GM-CSF were quantified using a Cytometric Bead Array Flex Set (BD) in a FACS Canto II flow cytometer. The detection limits were 1.25, 0.2, 1.2, and 0.2 pg/ml, respectively.

### IFN score

An IFN score was calculated as previously described by our group (15). Briefly, mRNA was isolated from whole blood and the gene expression of IFI44, IFI44L, MX1 and IFI6 was evaluated with TaqMan pre-designed assays by RT quantitative PCR. Expression levels were evaluated by the 2^−Δt^ method, using the GAPDH gene expression as housekeeping to normalize Ct values. The expression levels were log-transformed. Z-scores were calculated for each gene and an IFN score was calculated by averaging all genes per individual.

### Flow cytometry

BLyS surface expression (mBLyS) was quantified in 35 RA patients and 31 HC by flow cytometry on B-cells, neutrophils, monocytes and mDCs as previously described (12) using the following fluorescent-labeled antibodies: BLyS-FITC (eBioscience), CD19-CF-Blue (Immunostep), CD14-APC-Cy7 (BioLegend), CD1c-APC (eBioscience), or fluorochrome-matched control antibodies (all from eBioscence). Whole blood cells were extracellularly stained with the appropriate monoclonal antibodies or isotype controls for 30 min at 4°C. Then, red blood cells were lysed and cells were washed twice with PBS.

Then, cells were acquired and different leukocyte subpopulations were identified according to the expression of specific surface markers: mDCs (CD19-CD1c+), monocytes (CD14+), B-cells (CD19+). Neutrophils were identified based on their forward and side scatter signal. mBLyS expression was calculated as the mean fluorescence intensity (MFI) of the gated populations after subtracting the background signal from the respective isotype control.

### Statistical analyses

Continuous variables were summarized as median (interquartile range) or mean ± standard deviation. Categorical variables were expressed as n(%). Differences among groups were analyzed by Mann Withney U, Kruskal-Wallis (with Dunn-Bonferroni correction for multiple comparisons) or χ^2^ tests, as appropriate. Wilcoxon test was used for paired samples. Correlations were assessed by Spearman ranks' test. Cluster analysis was performed using squared euclidean distances and Ward's Minimum Variance Method, in order to identify clusters minimizing the loss of information (and minimize the effect of different sample sizes among clusters). R package heatmap.2 was used to generate the heatmap for visualization purposes. A *p* < 0.050 was considered as statistically significant. Statistical analyses were performed in SPSS 22.0, R 3.3.1 and GraphPad Prism 5.0. Sample size calculations and power analyses were carried out in PS–Power and Sample Size Calculations v. 3.1.2 (Vanderbilt University, United States).

## Results

### B-cell related factors in RA: differences between very early and established disease

**Figure 1:**
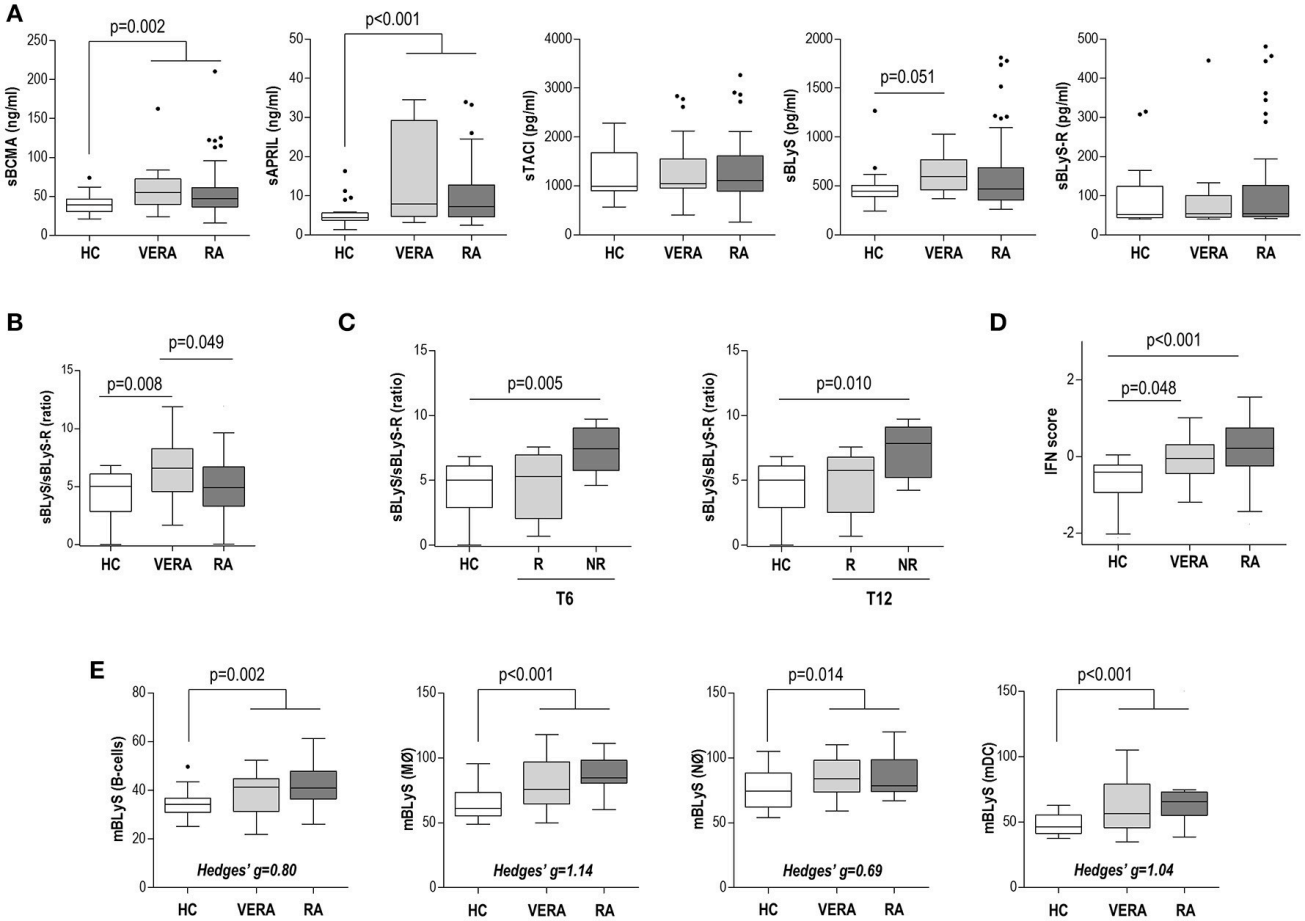
B-cell related factors in RA patients according to disease duration stage. **(A)** Serum levels of B-cell related factors and their decoy receptors in HC and RA patients classified as very early RA (VERA) or established RA (ERA). **(B)** Differences in the sBLyS/sBLyS-R ratio between HC, VERA and established RA patients. **(C)** Analysis of the sBLyS/sBLyS-R ratio in VERA patients depending on their clinical outcome at 6 (T6) or 12 (T12) months since disease onset. Patients were classified as responders (R) or non-responders (NR) according to EULAR criteria. **(D)** Analysis of the type I IFN score in HC and RA patients according to disease duration stage. **(E)** Analysis of the BLyS surface expression (mBLyS) on B-cells, monocytes (MØ), neutrophils (NØ) and myeloid DC (mDC). No differences between VERA and RA patients were observed. Hedges'g statistics were computed and added to the bottom of each graph to compare the size effect observed among cell populations. Boxes represent 25th and 75th percentiles, whereas whiskers represent minimum and maximum values. Statistical analyses were performed by Kruskal-Wallis with Dunn-Bonferroni tests for multiple comparisons **(A–D)** or Mann-Withney U tests **(E)**. P-values shown correspond to those obtained in the Dunn-Bonferroni tests **(A–D)** or Mann-Withney U-tests **(E)**.

The serum levels of sAPRIL, sBLyS, sBCMA, sTACI, and sBLyS-R were measured in 104 RA patients (including VERA and established RA) and 33 HC (Table 1). RA patients exhibited increased sAPRIL and sBCMA serum levels compared with HC, whereas no differences were observed for sTACI and sBLyS-R (Figure 1A). sBLyS was increased in VERA patients but not in patients with established disease (Figure 1A). The sBLyS levels in VERA were comparable, although slightly lower, to those observed in SLE patients (Supplementary Figure 1A). Interestingly, the sBLyS/sBLyS-R ratio was increased in VERA (Figure 1B). However, DAS28 was positively correlated to the sBLyS/sBLyS-R ratio (*r* = 0.298, *p* = 0.008) and negatively with sTACI (*r* = −0.315, *p* = 0.004) in established disease, but not in VERA. VERA patients were prospectively followed for 1 year and DAS28 score and clinical response to csDMARD treatment were registered. Of note, increased sBLyS/sBLyS-R ratio at onset was associated with a poor clinical response after 6 and 12 months (Figure 1C). No differences were observed for the rest of the B-cell factors analyzed.

**Table 1:** Characteristics of the subjects recruited in this study.

	**HC (*n* = 33)**	**RA (*n* = 104)**	***p*-value**
**DEMOGRAPHICAL FEATURES**			
Age, years; median (range)	49.16 (35.17–60.17)	54.37 (22.00–87.00)	0.104
Gender, f/m	25/8	85/19	0.452
**DISEASE FEATURES**			
Disease duration, years; median (range)		3.83 (0.00–15.00)	
Age at diagnosis, years; median (range)		49.37 (19.00–70.00)	
Disease activity (DAS28)		3.77 (2.02)	
Tender Joint Count		3.00 (7.00)	
Swollen Joint Count		2.00 (5.00)	
Patient Global Assessment (0-100)		45.00 (43.00)	
ESR, mm/h		18.00 (23.00)	
CRP, mg/l		2.22 (4.35)	
6 HAQ (0-3)		1.00 (1.22)	
RF (+), n (%)		60 (57.6)	
ACPA (+), n (%)		67 (64.4)	
Erosive disease, n (%)		40 (38.4)	
**TREATMENTS^a^, n (%)**			
Glucocorticoids		58 (67.4)	
Methotrexate		65 (75.5)	
TNFα blockers		32 (37.2)	
Tocilizumab		3 (3.4)	
**CYTOKINES SERUM LEVELS, MEAN ± SD**			
TNFα, pg/ml	79.78 ± 99.25	303.44 ± 234.83	<0.001
IFNα, pg/ml	2.28 ± 8.00	30.40 ± 70.55	0.002
MIP1α, pg/ml	1.37 ± 3.91	27.49 ± 52.17	<0.001
GM-CSF, pg/ml	1.85 ± 4.42	41.51 ± 47.15	<0.001
IFNg, pg/ml	4.14 ± 2.87	9.26 ± 17.66	0.020
IL-8, pg/ml	26.81 ± 33.26	54.51 ± 34.87	0.010
IL-10, pg/ml	1.33 ± 0.63	3.72 ± 11.66	0.006
IL-37, pg/ml	358.17 ± 1187.19	501.15 ± 1207.99	0.954
**IFN SCORE, MEAN ± SD**			
IFN score	−0.68 ± 0.71	0.23 ± 0.86	<0.001

a *Treatment usage was calculated as the number of patients using a given treatment among the number of patients receiving any treatment (n = 85)*.

Next, the associations between B-cell related factors and the IFN score were analyzed. IFN score was increased in VERA compared to HC and a greater increase was found in patients with established disease (Figure 1D). Interestingly, diverging associations were observed with the B-cell related factors: IFN score was negatively associated with sBLyS-R (*r* = −0.463, *p* = 0.031) in VERA patients, whereas it was positively associated with sBLyS-R (*r* = 0.271, *p* = 0.029) and sTACI (*r* = 0.210, *p* = 0.050) in established disease.

Finally, in order to shed new light into the BLyS production in RA, mBLyS surface expression was analyzed in different cell populations. RA patients exhibited an increased mBLyS expression on B-cells, neutrophils and, to a higher degree, on monocytes and mDCs (Figure 1E). No differences were observed between VERA and established RA. Both subsets of RA patients exhibited a similar mBLyS upregulation than that of found in SLE patients (Supplementary Figure 1B). Interestingly sTACI negatively correlated mBLyS expression on monocytes (*r* = −0.376, *p* = 0.037) and mDC (*r* = −0.465, *p* = 0.008) in HC, but not in RA. However, sBCMA was negatively associated with mBLyS expression on monocytes (*r* = −0.351, *p* = 0.030) in RA.

Overall, these results confirm that RA patients exhibit a complex picture of altered levels of B-cell related factors and decoy receptors, with important differences being found between the early and established stages of the disease. Different immune cell populations beyond B-cells, especially monocytes and mDCs, may be associated with the network of B-cell factors.

### B-cell factors profiling in RA: clinical and immunological relevance

**Figure 2:**
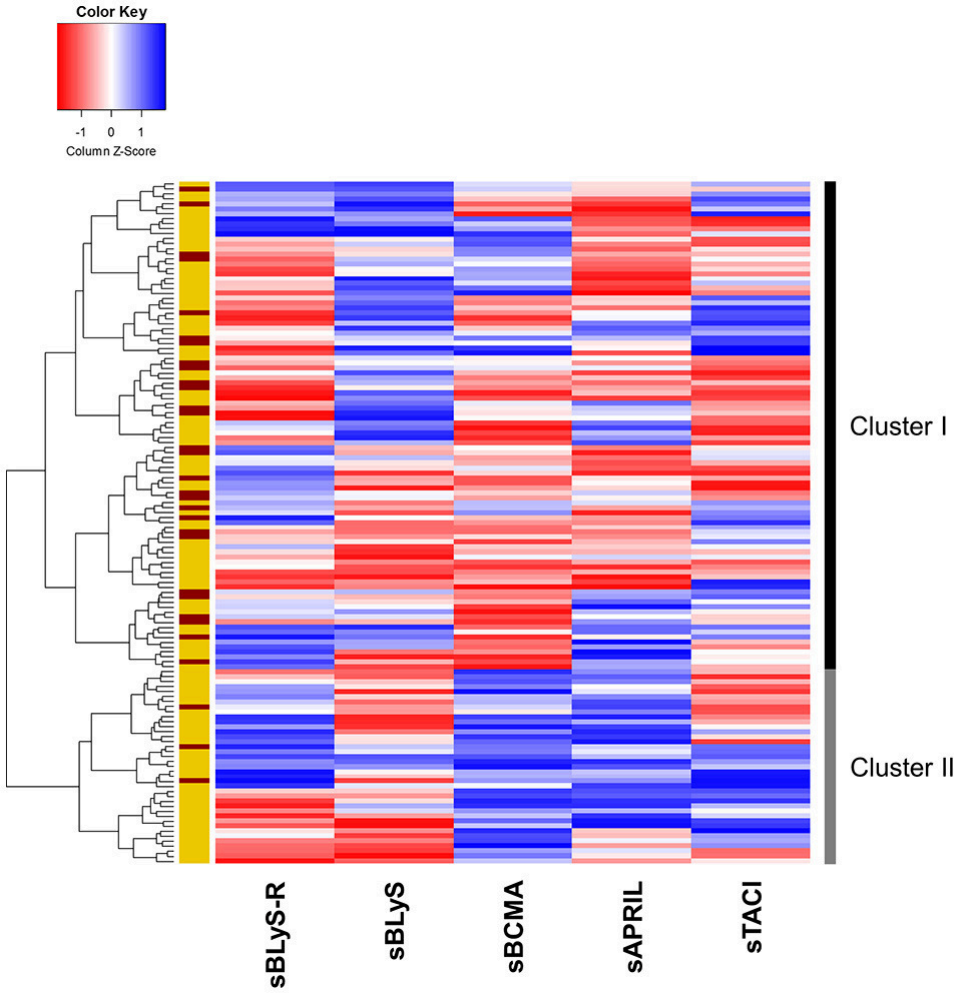
Cluster analysis of B-cell factors. Heatmap showing the dendrogram classification of the clusters based on the serum levels of B-cell factors and their decoy receptors (columns). Each row represents an individual. Colors in the vertical left bar denoted RA patients (yellow) and HC (red). Vertical right bar indicate the two clusters identified. Tiles are colored based on serum levels, red and blue indicating low or high levels, respectively.

In order to gain additional insight into the complex network of B-cell factors in RA, an integrative approach was performed. Then, an unsupervised cluster analysis based on sBLyS, sAPRIL, sBCMA, sTACI and sBLyS-R serum levels was conducted. Cluster analysis identified 2 clusters (thereafter referred to as I and II), cluster II being hallmarked by increased sAPRIL and sBCMA levels (Figure 2). Cluster II was overrepresented in RA compared to HC (32/104 vs. 3/33, *p* = 0.015). Sample sizes were confirmed to be adequate to identify the aforementioned clusters with the given frequencies at an alfa = 0.05.

Interestingly, the different profiles of B-cell factors were associated with clinical and immunological features in RA (Table 2). Cluster II-RA patients showed increased RF and ACPA positivity and were more likely to be treated with anti-TNFα agents compared to their cluster I-counterparts. Additionally, higher IFNα, MIP1α, TNFα, IL-37, GM-CSF serum levels, and IFN score were observed in patients of the cluster II. Apart from differences in serum levels, cluster II patients exhibited distinct associations of B-cell factors. sBLyS was positively correlated with DAS28 in cluster II-RA patients (*r* = 0.441, *p* = 0.013), but not in their cluster I counterparts. Moreover, sAPRIL levels exhibited a negative correlation with those of sBLyS-R (*r* = −0.463, *p* = 0.010) and sTACI (*r* = −0.411, *p* = 0.019) in cluster II-RA patients.

**Table 2:** Clinical and immunological features of RA patients depending on B-cell factors clusters.

	**Cluster I (*n* = 72)**	**Cluster II (*n* = 32)**	***p*-value**
**DEMOGRAPHICAL FEATURES**			
Age, years; median (range)	53.95 (22.00–79.92)	55.33 (22.42–87.00)	0.714
Gender, f/m	59/13	26/6	0.933
**DISEASE FEATURES**			
Disease duration, years; median (range)	3.12 (0.00–15.00)	3.98 (0.00–13.80)	0.937
Age at diagnosis, years; median (range)	49.37 (20.00–70.00)	49.29 (19.00–65.00)	0.743
Disease activity (DAS28)	3.75 (1.92)	4.05 (2.32)	0.729
Tender Joint Count	3.00 (7.00)	3.00 (2.00)	0.801
Swollen Joint Count	2.00 (5.00)	1.00 (5.00)	0.241
Patient Global Assessment (0-100)	48.00 (44.25)	40.00 (48.00)	0.142
ESR, mm/h	18.00 (21.25)	21.00 (40.50)	0.509
CRP, mg/l	2.07 (4.00)	2.65 (9.78)	0.281
HAQ (0-3)	1.00 (1.13)	1.00 (1.38)	0.837
RF (+), n (%)	36 (50.0)	24 (75.0)	**0.008**
ACPA (+), n (%)	39 (24.1)	28 (87.5)	**0.006**
Erosive disease, n (%)	26 (36.1)	14 (43.7)	0.562
**TREATMENTS ^a^, n (%)**			
Glucocorticoids	45 (72.5)	13 (54.1)	0.240
Methotrexate	47 (75.8)	18 (75.0)	0.938
TNFα blockers	20 (32.2)	12 (50.0)	**0.003**
Tocilizumab	1 (1.9)	2 (8.3)	0.179
**CYTOKINES SERUM LEVELS, MEAN ± SD**			
TNFα, pg/ml	247.81 ± 188.80	428.62 ± 279.68	**<0.001**
IFNα, pg/ml	11.55 ± 42.59	59.38 ± 103.93	**0.020**
MIP1α, pg/ml	17.78 ± 27.97	47.89 ± 80.98	**0.035**
GM-CSF, pg/ml	31.61 ± 30.01	63.76 ± 47.69	**<0.001**
IFNg, pg/ml	7.10 ± 14.43	13.77 ± 22.40	0.154
IL-8, pg/ml	51.20 ± 31.65	61.87 ± 40.72	0.404
IL-10, pg/ml	3.75 ± 13.66	3.63 ± 4.95	0.138
IL-37, pg/ml	257.35 ± 671.87	1055.99 ± 1841.74	**0.015**
**IFN SCORE, MEAN ± SD**			
IFN score	0.07 ± 0.81	0.63 ± 0.90	**0.015**

a *Treatment usage was calculated as the number of patients using a given treatment among the number of patients receiving any treatment (cluster I: n = 61, cluster II: n = 24). Statistical analyses with a p-value below 0.050 are highlighted in bold*.

All these results confirm that distinct subsets of RA patients can be distinguished based on B-cell factors. RA patients characterized by elevated sAPRIL and sBCMA levels exhibited a higher prevalence of autoantibodies, increased type I IFN activation and elevated levels of proinflammatory and immune-stimulatory mediators despite being largely treated with anti-TNFα agents.

### B-cell factors upon TNFα-blockade: association with clinical outcome

Due to the association between the B-cell profile and anti-TNFα usage, we decided to further investigate the changes in B-cell factors serum levels upon anti-TNFα therapy and their associations with the clinical response in a prospective study. To this aim, the serum levels of B-cell factors and mBLyS expression were evaluated in a group of 13 biological-naïve RA patients at baseline (BL) and after 3 months upon TNFα-blockade (PT, post-treatment).

**Figure 3:**
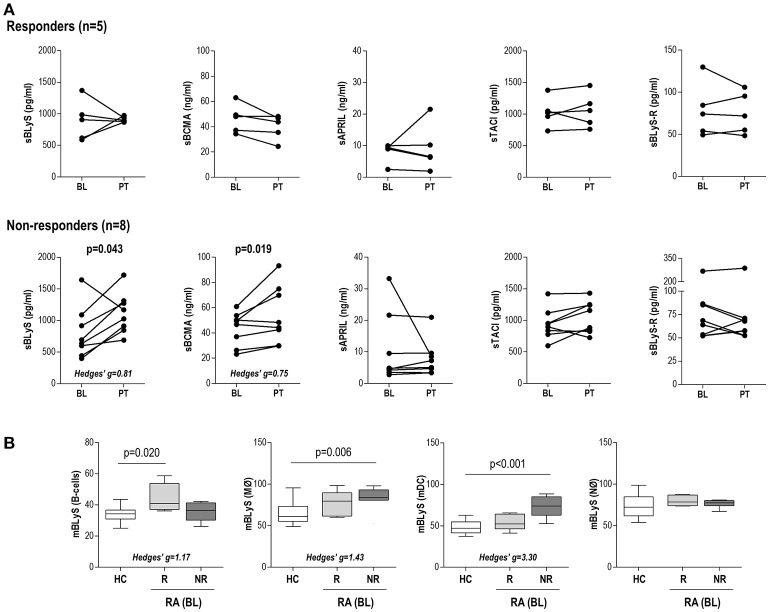
B-cell related factors upon TNFα-blockade in RA patients. **(A)** Paired analyses of the changes in the serum levels of B-cell related factors at baseline (BL) and post-treatment (PT) after TNFα-blockade in a subgroup of 13 RA patients prospectively followed. Patients were classified as responders or non-responders according to EULAR criteria. Each dot represents an individual. Statistical analysis was performed by Wilcoxon test. **(B)** Analysis of the BLyS surface expression (mBLyS) at baseline (BL) on B-cells, monocytes (MØ), neutrophils (NØ) and myeloid DC (mDC) after 3 months of TNFα-blockade. Patients were classified as responders (R) or non-responders (NR). Boxes represent 25th and 75th percentiles, whereas whiskers represent minimum and maximum values. Differences were assessed by Kruskal-Wallis with Dunn-Bonferroni tests for multiple comparisons tests. P-values shown correspond to those obtained in the multiple comparisons tests.

No changes after treatment were observed in any of the B-cell factors analyzed (sBCMA: *p* = 0.507, sAPRIL: *p* = 0.807, sTACI: *p* = 0.094, sBLyS: *p* = 0.116 and sBLyS-R: *p* = 0.701) in the whole group. However, when patients were stratified by EULAR clinical response, increasing sBCMA and sBLyS levels were found in those classified as non-responders (Figure 3A). No differences in baseline levels were observed between responders and non-responders. Importantly, no differences in demographical (age: *p* = 0.356 and gender: *p* = 0.580), clinical features (RF positivity: *p* = 0.529, ACPA positivity: *p* = 0.928) or treatments (glucocorticoid treatment: *p* = 0.836, glucocorticoid dosage: *p* = 0.209 and methotrexate dosage: *p* = 0.371) were found between responders and non-responders. Furthermore, prescription regiments were followed up by the patients, thus ruling out the possibility that adherence could be a confounding factor.

The analysis of the mBLyS expression upon TNFα-blockade revealed valuable insight into the relevance of different cell populations. First, sBLyS serum levels after treatment were positively correlated to mBLyS expression at baseline on monocytes (*r* = 0.391, *p* = 0.009), neutrophils (*r* = 0.359, *p* = 0.019) and mDC (*r* = 0.294, *p* = 0.050), whereas no association was observed with that of B-cells (*r* = 0.129, *p* = 0.405). Interestingly, these differences seem to mirror those of the levels of expression found among cell populations. On the other hand, the analysis of the mBLyS baseline expression levels according to the clinical response reinforced the differences observed among cell populations: whereas higher mBLyS on B-cells at baseline was associated with a good response, the opposite was observed when monocytes and mDCs were analyzed (Figure 3B). Finally, an integrative analysis including mBLyS expression on B-cells, monocytes and mDC was performed. The three variables were dichotomized using the Youden index (YI) of each distribution from COR curves to maximize specificity and sensitivity [B-cells: 36.25 (YI = 0.417), MO: 80.45 (YI = 0.708), and mDC: 61.70 (YI = 0.667)]. Next, the three categorized variables were entered in a logistic regression analyses together with potential confounders (age, gender and RF/ACPA positivity) to identify the main predictor(s) of clinical response. Using the Wald backwards method, only the categorized mBLyS expression on monocytes remained in the model as independent predictor (OR[95% CI]: 28.000[1.350, 108.591], *p* = 0.031).

Taken together, these results highlight a link between increasing levels of B-cell factors and a poor clinical outcome upon TNFα-blockade. A distinct contribution of different immune cell populations can be expected, monocytes playing a prominent effect.

## Discussion

B-cell activation represents a hallmark of RA pathogenesis. Unveiling the main mechanisms and mediators underlying this process is of outstanding relevance in RA and other rheumatic conditions, not only from the perspective of basic science but also to delineate potential therapeutic approaches. In the present manuscript, we described the altered levels of B-cell related factors and their decoy receptors in RA in relation to their potential clinical relevance. To the best of our knowledge, this is the first study providing a simultaneous analysis of different B-cell factors in soluble and membrane-bound isoforms together with the whole spectrum of their decoy receptors in RA. Additionally, a thorough study of their clinical relevance to treatment outcomes is prospectively addressed.

A major breakthrough of our study were the differences observed between the very early and the established disease stages. A clearly different role of the BLyS/BLyS-R axis was observed between these disease stages. Increased levels of BLyS in serum (20) and expression levels (21) were previously reported in VERA, with some controversy results being published elsewhere (22). However, our results went further by studying its associations with its highly specific soluble receptor and related activating factors, as well as prospectively analyzing its role as a biomarker of clinical outcome. On the contrary, not only sTACI was associated with disease activity in established RA, but a distinct pattern of B-cell factors was observed in these patients. These findings suggest that different pictures of B-cell factors are related to B-cell activation along RA the course. BLyS is mostly linked to naïve B-cell activation, whereas its role in antigen-experienced B-cell subsets is debated (23, 24). On the contrary, APRIL and BCMA govern the activation of the memory B-cell subsets as well as plasma cell differentiation and survival (25, 26). Importantly, APRIL supports plasma cell in the absence of BLyS (27). Moreover, BLyS-R expression is dominant in B-cells from germinal centers, whereas TACI and BCMA expression are characteristic of memory and short-lived antigen-producing cells (28). The association between APRIL and BCMA with ACPA/RF positivity observed in our study supports this notion. Then, it is likely that the very early stage of the disease could be strongly BLyS-dependent, whereas a different pattern of B-cell factors, mainly APRIL-dependent, underlie the chronic phase of the disease. This finding may challenge a previous study by Geng et al. (22), reporting elevated sBLyS levels in sero-negative RA independently of disease duration. However, the lack of information about the additional B-cell activating factors in their study did not allow to a proper comparison between studies. Taken together, these observations shed new light into the B-cell factor network in RA and may provide a rationale for treatment decision-making when considering B-cell factors as therapeutic targets.

Furthermore, our data showed a pronounced elevation of B-cell factors in RA, whereas their decoy receptors mostly remained within the normal range. Interestingly, the degree of occupancy of BLyS receptors in SLE has been related to disease activity (29). Importantly, our study demonstrates comparable sBLyS serum levels in RA and SLE patients. The decoy receptors efficiently block the soluble and membrane-bound forms of the B-cell factors (30, 31), hence suggesting that the analysis of the B-cell factors alone can provide limited information. However, the analysis of decoy receptors has been largely neglected in previous studies in RA. Our findings delineate a picture of elevated B-cell factors in RA with unchanged levels of its decoy receptors, hence pointing toward a less efficient negative feedback of the latter. This is supported by the findings on the ratio between sBLyS and sBLyS-R in our study, which may represent a surrogate marker of the degree of occupancy of sBLyS-R. Although a pivotal role of BLyS-R in the development of autoimmunity and antibody production was described in mice models (32), it had not previously evaluated in human patients until date.

Additionally, the analysis of the membrane expression of BLyS confirmed that different cell populations may account for the increased levels of B-cell factors in RA. Although classically linked to B-cell activation, the expression of BLyS and other B-cell related factors has been observed in other cell populations, such as neutrophils and monocytes (9, 10). However, whether this could be relevant to RA pathogenesis and extended to other cell populations remained unknown. The findings herein presented revealed a widespread BLyS expression beyond the B-cell compartment in RA, mainly on monocytes and mDCs subsets, linked to clinical outcomes. In fact, the mBLyS upregulation was similar to that of observed in SLE patients. More importantly, the membrane expression of BLyS seemed to have different clinical relevance depending on the cell population involved, especially concerning the clinical response to TNFα-blockade. On the one hand, increased BLyS expression on myeloid subsets was related to a poor clinical outcome, and BLyS expression levels at baseline on monocytes was positively correlated to sBLyS levels after treatment, which was in turn associated with a poor clinical response. This is especially relevant since sBLyS is the major contributor of its biological effects compared to the membrane-bound isoform, as evidenced in trans-well culture assays with B and myeloid cells (4, 33), and orchestrates the macrophage and dendritic cell-dependent regulation of human B-cell proliferation (34). Importantly, previous studies from our group have highlighted differences in BLyS mobilization in monocytes compared with other cell populations (11). On the other hand, increased BLyS on B-cells was found in RA patients exhibiting a good response. It is important to note that BLyS has been reported to show a protective effect in certain scenarios related to B-cell activation (35, 36), and a regulatory function of APRIL (37), presumably via a Breg-dependent mechanism (38), has been suggested in APRIL transgene mice. Interestingly, other APRIL transgene and knock out models showed yielded different conclusions [reviewed in (4)]. Taken together, the cell populations producing and reacting to BLyS emerge as key players to understand its clinical relevance, hence adding another layer of complexity to this scenario.

Our findings broaden the relevance of the B-cell factor network by documenting its association with myeloid cell subsets, key players of the innate immunity. In fact, severalinnate immune mediators were found to be elevated in patients with high levels of sAPRIL and sBCMA (cluster II), which may orchestrate the crosstalk between B-cell and innate subsets. Among them, the type I IFN must be remarked. Because of their immune-activation capacities, type I IFN have been extensively studied in systemic autoimmune conditions (39). Although type I IFN were known to prompt a B-cell over-activation, it was only in recent years that a link between IFN and BLyS was reported in different conditions (13, 40) and confirmed *in vitro* (41). Also, a similar effect was reported for APRIL (42). Our results showed for the first time an association between both IFN signature and B-cell factors in RA. A similar association was observed at the protein level with IFNα serum level, hence strengthening this finding. Moreover, although the TLR pathway had been associated with the sBCMA shedding from plasmacytoid DC (43), our results went further by reporting an association with other decoy receptors of the BLyS/APRIL system. Interestingly, the association between the IFN score and sBLyS-R and sTACI was dependent on the disease stage, which is in line with the heterogeneity of the IFN score reported in RA along the disease course (15), as well as with the differential associations of sBLyS previously observed. In fact, not all the IFN-responding genes are equally linked to BLyS [data not shown and (44)]. Therefore, a detailed analysis of the IFN-responding genes in relation to BLyS expression warrants further studies.

B-cells are pivotal therapeutic targets in RA, as evidenced by early studies with rituximab (45). However, rituximab treatment does not affect all B-cell subsets and changes on B-cell frequency and IgG levels do not always parallel clinical response (2), thus underlining the involvement of additional mediators. However, B-cell factors exhibit complex and mutual mechanisms of regulation, with certain degree of overlap and even redundancy, as observed in our study. Indeed, not all B-cell factors are equally affected by rituximab treatment (46). The differential effect of belimumab (which targets soluble BLyS) and tabalumab (which targets both soluble and membrane-bound isoforms) supports this notion. More importantly, B-cell factors are usually present in serum in heteromeric isoforms whose composition is thought to play a significant role (47, 48), and the decoy receptors are known to effectively block heteromeric complexes (48), although to a variable degree (49). Therefore, a therapeutic approach solely based on a single B-cell factor may not be appropriate in this scenario, as demonstrated by the modest, albeit significant, the clinical effect of anti-BLyS therapies in RA [reviewed in (16)], in contrast to SLE, where BLyS plays a prominent pathogenic role. As a consequence, additional mediators are thought to be differentially involved in RA, hence bringing the attention to the B-cell factor network in this conditions. This led to the development of a combined approach to target both BLyS and APRIL (atacicept). The growing list of biological therapies together with theirelevated costs strongly stress the need for biomarkers to assist in patient stratification to ensure cost-effectiveness of such treatments. Recent results from AUGUST trials (I and II) revealed a clear biological activity of this drug, whereas the clinical endpoint was not achieved (50, 51), thus emphasizing the need of a personalized approach to assist in patient stratification. In this sense, we have conducted an unsupervised, hypothesis-generating integrative approach to identify subsets of patients based on their profiles of B-cell factors. Those patients with increased levels of these factors can be considered as those more likely to respond to such interventions. Our results revealed that increased sAPRIL and sBCMA identify a subset of patients with an increased prevalence of autoantibodies, and a pronounced proinflammatory milieu despite being largely treated with anti-TNFα drugs, which points to a poor response to these agents, as confirmed in our prospective analysis. Together with the increased expression of mBLyS observed in RA patients, our results may provide a rationale for decision-making in atacicept prescription. Not only by blocking elevated APRIL serum levels but also by removing circulating myeloid cells with increased mBLyS expression, atacicept could represent a promising therapy in these patients. Of note, a *post-hoc* analysis of the APRIL-SLE study demonstrated the usefulness of BLyS and APRIL serum levels as biomarkers to predict clinical response to atacicept (52), linked to a greater, dose-dependent pharmacodynamics effect. Moreover, patients with elevated levels of both APRIL and BLyS exhibited a more severe disease, which is line with our findings in RA patients.

In conclusion, our study demonstrates an upregulation of B-cell factors in both serum and membrane-bound forms in RA patients depending on their disease stage, together with unchanged levels of their decoy receptors. Myeloid cell populations seem to contribute to the BLyS overexpression in RA. Moreover, sAPRIL and sBCMA serum levels identify a subset of patients with a more severe disease and increased prevalence of autoantibodies, probably linked to a B-cell over-activation and immune-stimulatory status. Finally, poor response to TNFα-blockade was associated with increasing B-cell factors. Overall, an integrative approach of the network of B-cell factors may assist in patient stratification. Finally, it is important to consider that this work represents a proof-of-concept study on the B-cell factors network and its clinical relevance in RA. As such, a number of limitations must be remarked, like the observational design and the reduced sample size of some analyses. Further follow-up studies are needed to elucidate the clinical benefit of these findings in the long-term. Also, separate analyses on individual cell populations will shed new light into the biological mechanisms underlying the findings herein described.

## Author contributions

JR-C performed most of the experimental procedures, carried out the statistical analyses and drafted and edited the manuscript. MA-L and FB-G were in charge of patients' recruitment and clinical data collection. PL performed some experimental procedures. AS conceived the study, designed the protocols and edited the manuscript. All authors read and approved the final version of the manuscript.

### Conflict of interest statement

The authors declare that the research was conducted in the absence of any commercial or financial relationships that could be construed as a potential conflict of interest.

## Abbreviations

ACPA: anti-citrullinated peptide antibodies
APRIL: A Proliferation-Inducing Ligand
BCMA: B-cell Maturation Antigen
BLyS: B Lymphocyte Stimulating factor
CRP: C-reactive protein
csDMARDs: conventional synthetic DMARDs
DAS28: disease activity score 28-joints
DMARDs: disease-modifying antirheumatic drugs
EULAR: European league against rheumatism
ESR: erythrocyte sedimentation rate
HAQ: health assessment questionnaire
HC: healthy controls
IFI6: interferon alpha inducible protein 6
IFI44: interferon induced protein 44
IFI44L: interferon induced protein 44 like
IFN: interferon
IRG: interferon-responding gene
MHC: major histocompatibility factor
MX1: MX dynamin like GTPase 1
NSAIDs: non-steroidal anti-inflammatory drugs
RA: rheumatoid arthritis
RF: rheumatoid factor
TACI: Transmembrane Activator and CALM Interactor
TNFα: tumor necrosis factor alpha.

## References

[1] Bugatti S Codullo V Caporali R Montecucco C. B cells in rheumatoid arthritis. Autoimmun Rev. (2007) 7:137–42. 10.1016/j.autrev.2007.02.017 18035324

[2] McInnes IB Schett G. The pathogenesis of rheumatoid arthritis. N Engl J Med. (2011) 365:2205–19. 10.1056/NEJMra1004965 22150039

[3] Seyler TM Park YW Takemura S Bram RJ Kurtin PJ Goronzy JJ. BLyS and APRIL in rheumatoid arthritis. J Clin Invest. (2005) 115:3083–92. 10.1172/JCI25265 16239971 PMC1257539

[4] Baker KP. BLyS–an essential survival factor for B cells: basic biology, links to pathology and therapeutic target. Autoimmun Rev. (2004) 3:368–75. 10.1016/j.autrev.2004.02.001 15288003

[5] Pers JO Daridon C Devauchelle V Jousse S Saraux A Jamin C. BAFF overexpression is associated with autoantibody production in autoimmune diseases. Ann N Y Acad Sci. (2005) 1050:34–9. 10.1196/annals.1313.004 16014518

[6] Binard A Le Pottier L Saraux A Devauchelle-Pensec V Pers J-O Youinou P. Does the BAFF dysregulation play a major role in the pathogenesis of systemic lupus erythematosus? J Autoimmun. 30:63–7. 10.1016/j.jaut.2007.11.001 18155417

[7] Day ES Cachero TG Qian F Sun Y Wen D Pelletier M. Selectivity of BAFF/BLyS and APRIL for binding to the TNF family receptors BAFFR/BR3 and BCMA. Biochemistry. (2005) 44:1919–31. 10.1021/bi048227k 15697217

[8] Thompson JS Bixler SA Qian F Vora K Scott ML Cachero TG. BAFF-R, a newly identified TNF receptor that specifically interacts with BAFF. Science (2001) Sep 14;293:2108–11. 10.1126/science.1061965 11509692

[9] Scapini P Bazzoni F Cassatella MA. Regulation of B-cell-activating factor (BAFF)/B lymphocyte stimulator (BLyS) expression in human neutrophils. Immunol Lett. (2008) 116:1–6. 10.1016/j.imlet.2007.11.009 18155301

[10] Nardelli B Belvedere O Roschke V Moore PA Olsen HS Migone TS. Synthesis and release of B-lymphocyte stimulator from myeloid cells. Blood (2001) 97:198–204. 10.1182/blood.V97.1.198 11133761

[11] López P Scheel-Toellner D Rodríguez-Carrio J Caminal-Montero L Gordon C Suárez A. Interferon-α-induced B-lymphocyte stimulator expression and mobilization in healthy and systemic lupus erthymatosus monocytes. Rheumatology (Oxford) (2014) 53:2249–58. 10.1093/rheumatology/keu249 24942493

[12] López P Rodríguez-Carrio J Caminal-Montero L Mozo L Suárez A. A pathogenic IFNα, BLyS and IL-17 axis in systemic lupus erythematosus patients. Sci Rep. (2016) 6:20651. 10.1038/srep20651 26847824 PMC4742957

[13] Brkic Z Maria NI van Helden-Meeuwsen CG van de Merwe JP van Daele PL Dalm VA. Prevalence of interferon type I signature in CD14 monocytes of patients with Sjogren's syndrome and association with disease activity and BAFF gene expression. Ann Rheum Dis. (2013) 72:728–35. 10.1136/annrheumdis-2012-201381 22736090 PMC3618683

[14] Brkic Z van Bon L Cossu M van Helden-Meeuwsen CG Vonk MC Knaapen H. The interferon type I signature is present in systemic sclerosis before overt fibrosis and might contribute to its pathogenesis through high BAFF gene expression and high collagen synthesis. Ann Rheum Dis. (2016) 75:1567–73. 10.1136/annrheumdis-2015-207392 26371289

[15] Rodríguez-Carrio J Alperi-López M López P Ballina-García FJ Suárez A. Heterogeneity of the type I interferon signature in rheumatoid arthritis: a potential limitation for its use as a clinical biomarker. Front Immunol. (2017) 8:2007. 10.3389/fimmu.2017.02007 29387065 PMC5775969

[16] Richez C Truchetet ME Schaeverbeke T Bannwarth B. Atacicept as an investigated therapy for rheumatoid arthritis. Expert Opin Investig Drugs (2014) 23:1285–94. 10.1517/13543784.2014.943835 25078871

[17] Smolen JS Landewé R Bijlsma J Burmester G Chatzidionysiou K Dougados M. EULAR recommendations for the management of rheumatoid arthritis with synthetic and biological disease-modifying antirheumatic drugs: 2016 update. Ann Rheum Dis. (2017) 76:960–77. 10.1136/annrheumdis-2016-210715 28264816

[18] Van Gestel AM Anderson JJ Van Riel PLCM Boers M Haagsma CJ Rich B ACR and EULAR improvement criteria have comparable validity in rheumatoid arthritis trials. J Rheumatol. (1999) 26:705–11. 10090187

[19] Hochberg MC. Updating the American College of Rheumatology revised criteria for the classification of systemic lupus erythematosus. Arthritis Rheum. (1997) 40:1725. 9324032 10.1002/art.1780400928

[20] Moura RA Cascão R Perpétuo I Canhão H Vieira-Sousa E Mourão AF. Cytokine pattern in very early rheumatoid arthritis favours B-cell activation and survival. Rheumatology (Oxford) (2011) 50:278–82. 10.1093/rheumatology/keq338 21047805

[21] Moura RA Canhão H Polido-Pereira J Rodrigues AM Navalho M Mourão AF. BAFF and TACI gene expression are increased in patients with untreated very early rheumatoid arthritis. J Rheumatol. (2013) 40:1293–302. 10.3899/jrheum.121110 23772083

[22] Geng Y Zhang Z. Comparative study on the level of B lymphocyte stimulator (BlyS) and frequency of lymphocytes between sero-negative and sero-positive rheumatoid arthritis patients. Int J Rheum Dis. (2012) 15:478–85. 10.1111/j.1756-185X.2012.01814.x 23083038

[23] Scholz JL Crowley JE Tomayko MM Steinel N O'Neill PJ Quinn WJ. BLyS inhibition eliminates primary B cells but leaves natural and acquired humoral immunity intact. Proc Natl Acad Sci USA. (2008) 105:15517–22. 10.1073/pnas.0807841105 18832171 PMC2563088

[24] Treml LS Carlesso G Hoek KL Stadanlick JE Kambayashi T Bram RJ. TLR stimulation modifies BLyS receptor expression in follicular and marginal zone B cells. J Immunol. (2007) Jun 15; 178:7531–9. 10.4049/jimmunol.178.12.7531 17548587

[25] Belnoue E Pihlgren M McGaha TL Tougne C Rochat A-F Bossen C. APRIL is critical for plasmablast survival in the bone marrow and poorly expressed by early-life bone marrow stromal cells. Blood (2008) 111:2755–64. 10.1182/blood-2007-09-110858 18180376

[26] Castigli E Scott S Dedeoglu F Bryce P Jabara H Bhan AK. Impaired IgA class switching in APRIL-deficient mice. Proc Natl Acad Sci USA. (2004) 101:3903–8. 10.1073/pnas.0307348101 14988498 PMC374342

[27] Benson MJ Dillon SR Castigli E Geha RS Xu S Lam KP. Cutting edge: the dependence of plasma cells and independence of memory B cells on BAFF and APRIL. J Immunol. (2008) 180:3655–9. 10.4049/jimmunol.180.6.3655 18322170

[28] Driver DJ McHeyzer-Williams LJ Cool M Stetson DB McHeyzer-Williams MG. Development and maintenance of a B220- memory B cell compartment. J Immunol. (2001) 167:1393–405. 10.4049/jimmunol.167.3.1393 11466358

[29] Carter RH Zhao H Liu X Pelletier M Chatham W Kimberly R. Expression and occupancy of BAFF-R on B cells in systemic lupus erythematosus. Arthritis Rheum. (2005) 52:3943–54. 10.1002/art.21489 16320342

[30] Laurent SA Hoffmann FS Kuhn PH Cheng Q Chu Y Schmidt-Supprian M. γ-Secretase directly sheds the survival receptor BCMA from plasma cells. Nat Commun. (2015) 6:7333. 10.1038/ncomms8333 26065893 PMC4490565

[31] Ware CF. Decoy receptors thwart B cells. Nature (2000) 404:949–50. 10.1038/35010263 10801112

[32] Ju ZL Shi GY Zuo JX Zhang JW Jian Sun. Unexpected development of autoimmunity in BAFF-R-mutant MRL-lpr mice. Immunology (2007) 120:281–9. 10.1111/j.1365-2567.2006.02500.x 17073941 PMC2265857

[33] Schneider P MacKay F Steiner V Hofmann K Bodmer JL Holler N. BAFF, a novel ligand of the tumor necrosis factor family, stimulates B cell growth. J Exp Med. (1999) 189:1747–56. 10.1084/jem.189.11.1747 10359578 PMC2193079

[34] Craxton A Magaletti D Ryan EJ Clark EA. Macrophage- and dendritic cell–dependent regulation of human B-cell proliferation requires the TNF family ligand BAFF. Blood (2003) 101:4464–71. 10.1182/blood-2002-10-3123 12531790

[35] Jackson SW Scharping NE Jacobs HM Wang S Chait A Rawlings DJ. Cutting edge: BAFF overexpression reduces atherosclerosis via TACI-dependent B cell activation. J Immunol. (2016) 197:4529–34. 10.4049/jimmunol.1601198 27837104 PMC5147509

[36] Ponnuswamy P Joffre J Herbin O Esposito B Laurans L Binder CJ. Angiotensin II synergizes with BAFF to promote atheroprotective regulatory B cells. Sci Rep. (2017) 7:4111. 10.1038/s41598-017-04438-6 28646220 PMC5482806

[37] Fernandez L Salinas GF Rocha C Carvalho-Pinto CE Yeremenko N Papon L. The TNF family member APRIL dampens collagen-induced arthritis. Ann Rheum Dis. (2013) 72:1367–74. 10.1136/annrheumdis-2012-202382 23178293

[38] Hua C Audo R Yeremenko N Baeten D Hahne M Combe B. A proliferation inducing ligand (APRIL) promotes IL-10 production and regulatory functions of human B cells. J Autoimmun. (2016) 73:64–72. 10.1016/j.jaut.2016.06.002 27372914

[39] Rönnblom L Eloranta ML. The interferon signature in autoimmune diseases. Curr Opin Rheumatol. (2013) 25:248–53. 10.1097/BOR.0b013e32835c7e32 23249830

[40] Jacob N Guo S Mathian A Koss MN Gindea S Putterman C. B Cell and BAFF dependence of IFN-α-exaggerated disease in systemic lupus erythematosus-prone NZM 2328 mice. J Immunol. (2011) 186:4984–93. 10.4049/jimmunol.1000466 21383240 PMC3074466

[41] Ittah M Miceli-Richard C Eric Gottenberg J Lavie F Lazure T Ba N. B cell-activating factor of the tumor necrosis factor family (BAFF) is expressed under stimulation by interferon in salivary gland epithelial cells in primary Sjögren's syndrome. Arthritis Res Ther. (2006) 8:R51. 10.1186/ar1912 16507175 PMC1526588

[42] Hardenberg G Planelles L Schwarte CM van Bostelen L Le Huong T Hahne M. Specific TLR ligands regulate APRIL secretion by dendritic cells in a PKR-dependent manner. Eur J Immunol. (2007) 37:2900–11. 10.1002/eji.200737210 17899538

[43] Schuh E Musumeci A Thaler FS Laurent S Ellwart JW Hohlfeld R. Human plasmacytoid dendritic cells display and shed B cell maturation antigen upon TLR engagement. J Immunol. (2017) 198:3081–8. 10.4049/jimmunol.1601746 28283566

[44] Sjöstrand M Johansson A Aqrawi L Olsson T Wahren-Herlenius M Espinosa A. The expression of BAFF is controlled by IRF transcription factors. J Immunol. (2016) 196:91–6. 10.4049/jimmunol.1501061 26590315 PMC4683359

[45] Edwards JCW Szczepanski L Szechinski J Filipowicz-Sosnowska A Emery P Close DR. Efficacy of B-cell-targeted therapy with rituximab in patients with rheumatoid arthritis. N Engl J Med. (2004) 350:2572–81. 10.1056/NEJMoa032534 15201414

[46] Vallerskog T Heimbürger M Gunnarsson I Zhou W Wahren-Herlenius M Trollmo C. Differential effects on BAFF and APRIL levels in rituximab-treated patients with systemic lupus erythematosus and rheumatoid arthritis. Arthritis Res Ther. (2006) 8:R167. 10.1186/ar2076 17092341 PMC1794511

[47] Roschke V Sosnovtseva S Ward CD Hong JS Smith R Albert V. BLyS and APRIL form biologically active heterotrimers that are expressed in patients with systemic immune-based rheumatic diseases. J Immunol. (2002) 169:4314–21. 10.4049/jimmunol.169.8.4314 12370363

[48] Dillon SR Harder B Lewis KB Moore MD Liu H Bukowski TR. B-lymphocyte stimulator/a proliferation-inducing ligand heterotrimers are elevated in the sera of patients with autoimmune disease and are neutralized by atacicept and B-cell maturation antigen-immunoglobulin. Arthritis Res Ther. (2010) 12:R48. 10.1186/ar2959 20302641 PMC2888197

[49] Stohl W Hilbert DM. The discovery and development of belimumab: the anti-BLyS-lupus connection. Nat Biotechnol. (2012) 30:69–77. 10.1038/nbt.2076 22231104 PMC3264947

[50] Genovese MC Kinnman N de La Bourdonnaye G Pena Rossi C Tak PP. Atacicept in patients with rheumatoid arthritis and an inadequate response to tumor necrosis factor antagonist therapy: results of a phase II, randomized, placebo-controlled, dose-finding trial. Arthritis Rheum. (2011) 63:1793–803. 10.1002/art.30373 21452293

[51] van Vollenhoven RF Kinnman N Vincent E Wax S Bathon J Atacicept in patients with rheumatoid arthritis and an inadequate response to methotrexate: results of a phase II, randomized, placebo-controlled trial. Arthritis Rheum. (2011) 63:1782–92. 10.1002/art.30372 21452294

[52] Gordon C Wofsy D Wax S Li Y Pena Rossi C Isenberg D. Post hoc analysis of the phase II/III APRIL-SLE study: association between response to atacicept and serum biomarkers including BLyS and APRIL. Arthritis Rheumatol. (2017) 69:122–30. 10.1002/art.39809 27390168 PMC6681010

