# Supplementary material for: Profiling of B-Cell Factors and Their Decoy Receptors in Rheumatoid Arthritis: Association With Clinical Features and Treatment Outcomes
Source: Front Immunol. 2018 Oct 11;9:2351. doi: 10.3389/fimmu.2018.02351 (PMC6194314; doi:10.3389/fimmu.2018.02351)
Supplement: Supplementary file 1 [file Table_1.docx]

**SUPPLEMENTARY MATERIAL**

**Supplementary Table 1: Demograhic and clinical parameters of SLE patients entered in this study.**

|  | **SLE patients**  **(n=42)** |
| --- | --- |
| **Demographic features** |  |
| Gender, n female/male | 40/2 |
| Age, years (median (range)) | 49.6 (29.00 – 74.30) |
|  |  |
| **Clinical manifestations, n(%)** |  |
| Age at diagnosis, years (median (range)) | 31.50 (18.00 – 64.00) |
| Disease duration, years (mean ± SD) | 14.37 ± 9.24 |
| SLEDAI score (mean ± SD) | 3.65 ± 3.13 |
| Clinical criteria |  |
| Malar rash | 25 (59.5) |
| Discoid lesions | 8 (19.5) |
| Photosensitivity | 19 (45.2) |
| Oral ulcers | 25 (59.5) |
| Arthritis | 33 (78.6) |
| Serositis | 8 (19.0) |
| Cytopenia | 29 (69.0) |
| Renal disorder | 10 (23.8) |
| Neurological disorder | 4 (9.50) |
| **Autoantibodies, n(%)** |  |
| ANA | 120 (100.0) |
| Anti-dsDNA | 34 (81.0) |
| Anti-SSA | 17 (41.5) |
| Anti-SSB | 4 (9.8) |
| Anti-Sm | 4 (9.8) |
| Anti-RNP | 5 (12.2) |
| Rheumatoid factor | 5 (22.7) |
| Anti-cardiolipin IgG | 3 (7.1) |
| Anti-cardiolipin IgM | 5 (11.9) |
| **Treatments, n(%)** |  |
| None or NSAID | 2 (4.7) |
| Antimalarial drugs | 38 (90.5) |
| Glucocorticoids | 19 (45.2) |
| Immunosuppressive drugs | 1 (2.30) |

**Supplementary Figure Legends**

**Supplementary Figure 1:** (A) Levels of sBLyS in HC, RA patients classified as very early RA (VERA) or established RA (RA), and SLE patients (Supplementary Table 1). (B) Expression of mBLyS B-cells, monocytes (MØ), neutrophils (NØ) and myeloid DC (mDC) in HC, RA patients (VERA and RA) and SLE patients. No differences between VERA and RA patients were noted (see Figure 1). Boxes represent 25th and 75th percentiles, whereas whiskers represent minimum and maximum values. Statistical analyses were performed by Kruskal-Wallis with Dunn-Bonferroni tests for multiple comparisons. P-values shown correspond to those obtained in the Dunn-Bonferroni tests.
