# Supplementary figures and images for: Profiling of B-Cell Factors and Their Decoy Receptors in Rheumatoid Arthritis: Association With Clinical Features and Treatment Outcomes
Source: Front Immunol. 2018 Oct 11;9:2351. doi: 10.3389/fimmu.2018.02351 (PMC6194314; doi:10.3389/fimmu.2018.02351)

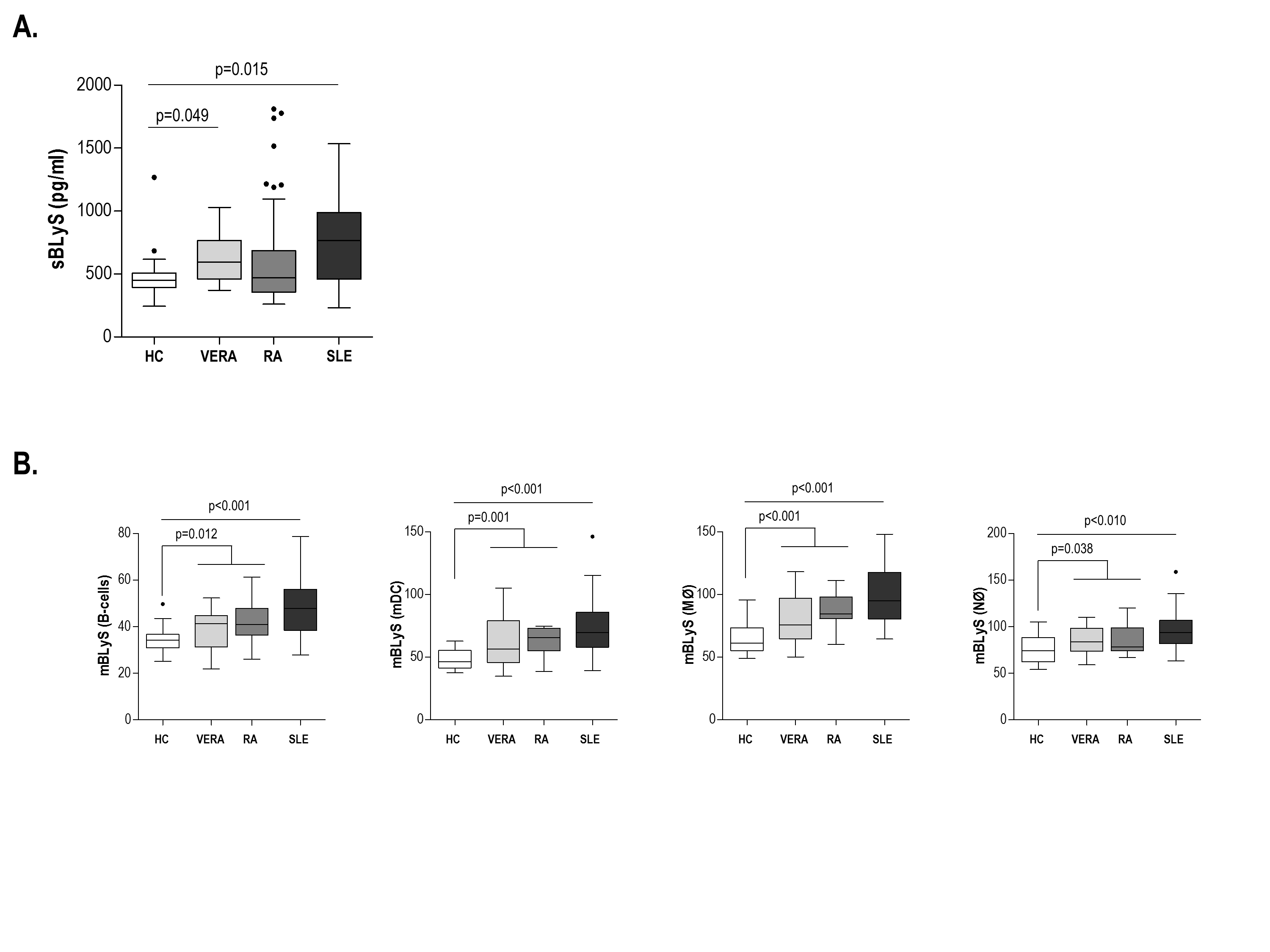

Supplement: Supplementary file 2 [file Image_1.TIF]
